# Late dysfunction of a mechanical aortic valve after long-term low molecular weight heparin therapy: a case report

**DOI:** 10.1093/ehjcr/ytae361

**Published:** 2024-07-27

**Authors:** Aye Mon, Selvaraj Shanmuganathan, Akhlaque Uddin

**Affiliations:** Department of Cardiology, Trent Cardiac Center, Nottingham City Hospital, Nottingham University Hospitals NHS Trust, Nottingham, UK; Department of Cardiothoracic Surgery, Trent Cardiac Center, Nottingham City Hospital, Nottingham University Hospitals NHS Trust, Nottingham, UK; Department of Cardiology, Trent Cardiac Center, Nottingham City Hospital, Nottingham University Hospitals NHS Trust, Nottingham, UK

**Keywords:** Mechanical aortic valve, Anticoagulation, Low molecular weight heparin, Warfarin intolerance, Bleeding, Thromboembolism, Redo valve surgery, Case report

## Abstract

**Background:**

To date, vitamin K anticoagulants are the only recommended long-term therapy for mechanical heart valves. Bleeding episodes, thromboembolic events, and international normalized ratio monitoring are difficult and prevalent complications for these patients. This report reflects the late mechanical aortic valve dysfunction after long-term low molecular weight heparin therapy.

**Case summary:**

A 66-year-old male patient underwent mechanical aortic valve replacement in 2007. He was administered therapeutic doses of enoxaparin for nearly 12 years due to warfarin-related bleeding complications and labile international normalized ratios. However, he experienced multiple cardiovascular and cerebrovascular thromboembolic events, including an anterolateral ST-elevation myocardial infarction with left anterior descending artery thrombus, treated with thrombus aspiration and stenting. The patient was eventually admitted with symptoms and signs of acute heart failure, and echocardiography, fluoroscopy, and a cardiac computed tomography detected mechanical aortic valve prosthesis dysfunction, with an immobile leaflet and pannus. The patient demonstrated no improvement despite switching to unfractionated heparin, and he ultimately underwent redo aortic bioprosthetic valve surgery with a favourable outcome.

**Discussion:**

Low molecular weight heparin is prescribed for patients with aortic mechanical valves who are intolerant to vitamin K antagonists or as bridging in certain situations. Anti-Xa factor monitoring should be considered for long-term prescriptions.

**Learning points**

- Vitamin K antagonists are the only approved anticoagulants to date for mechanical valve thrombosis prevention.
- The role of multimodality imaging is crucial in the diagnosis and therapeutic planning of mechanical heart valve thrombosis.
- The redo valve replacement is an extremely effective treatment option despite its associated surgical risks.

## Introduction

Mechanical valve replacement is recommended for those aged <60 years in the aortic position [Class IIa, European Society of Cardiology (ESC) recommendation]. The annual incidence rate of prosthetic valve thrombosis is 0.3–1.3%.^1^ The rate of major embolism without an antithrombotic treatment is 4/100 patient-years, and the rate of thromboembolism is four times lower in those receiving vitamin K antagonists (VKAs) (warfarin, acenocoumarin, phenprocoumon, and phenindione).^2^ Vitamin K antagonists are dose adjusted to maintain an international normalized ratio (INR) of 2–3 in mechanical aortic valves. The bleeding and thromboembolic complications among unfractionated heparin (UFH), low molecular weight heparin (LMWH), and warfarin are similar.^3^ The LMWH in patients contraindicated to VKAs is recommended for the first and third trimesters of pregnancy (ESC guideline 2011), but only a few studies evaluated the long-term use of this strategy. Human data regarding new oral anticoagulants in mechanical valves are limited, and only two small clinical trials revealed that rivaroxaban achieved protection against thromboembolic events in patients with mechanical heart valves (PROACTXA).^4^ Antiplatelets could not sufficiently prevent thrombosis in St. Jude mechanical aortic valves.^5^ Only two case reports of patients without any anticoagulation who survived for years without thromboembolic events have been presented.^6^

The patient in our case was intolerant to warfarin, with non-compliance issues. He was then prescribed LMWH for 12 years post-valve replacement with multiple bleeding and thromboembolic events, which eventually required redo tissue aortic valve surgery.

## Summary figure

**Table:** 

Date	Events
July 2007	Mechanical AVR and aortic root replacement with re-implantation of coronary arteries in supravalvular position for aortic incompetence and ascending aortic aneurysm
March 2008	Commenced treatment dose LMWH (enoxaparin) due to warfarin-related bleeding complications and erratic INR
July 2010	Non-ST-elevation myocardial infarction due to likely transient coronary artery thrombus, coronary angiogram showed unobstructed coronary arteries.
September 2010	Right-sided anterior circulation stroke
August 2011	Right frontal lobe infarction
December 2013	Left partial anterior circulation stroke
July 2017	Anterolateral ST-elevation myocardial infarction treated with aspiration thrombectomy followed by percutaneous coronary angioplasty with one stent for long segment of thrombus extended from left main stem to left anterior descending artery
April 2019	Heart failure and thrombus in the metallic aortic valve prosthesis noted on fluoroscopy and cardiac computed tomography.
June 2019	Failed conservative treatment with IV infusion UFH and underwent redo tissue AVR with favourable outcome.

## Case presentation

**Figure 1:**
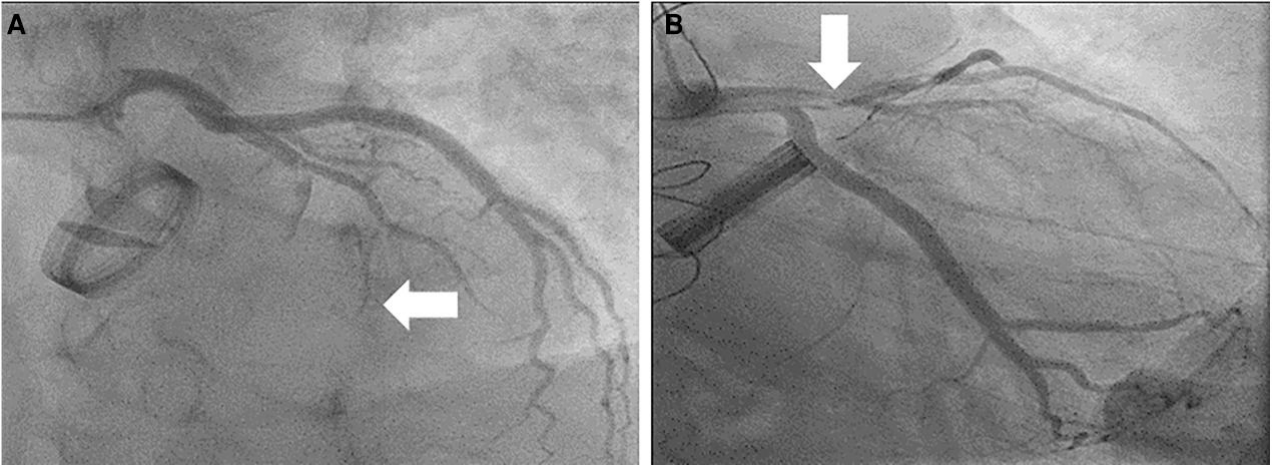
Coronary arteriogram in (*A*) posteroanterior cranial and (*B*) caudal view showing thrombus from the left main stem into occluded left anterior descending artery (white arrow).

A 66-year-old non-smoker male patient with a medical history of Type II diabetes, ischaemic heart disease, and four non-ST-elevation myocardial infarctions (NSTEMIs) from 2005 to 2007. In July 2007, he underwent mechanical aortic valve (27 mm CaroSeal) and root replacement with coronary artery re-implantation in the supravalvular position for aortic incompetence and an ascending aortic aneurysm, which may be syphilitic in origin. Over the last 12 years, the patient had not tolerated warfarin due to side effects and compliance issues and thus was managed with LMWH, enoxaparin of 1 mg/kg twice daily. He desired to undergo further surgery to replace his mechanical valve with a tissue valve and discussed this with the cardiac surgeons but was advised against further redo cardiac surgery due to the surgical risks and because he was asymptomatic. He experienced multiple thromboembolic episodes, including NSTEMI in July 2010, a right-sided anterior circulation stroke in September 2010, a further right frontal lobe infarction in August 2011, and a left partial anterior circulation stroke in December 2013. He presented with anterolateral STEMI in July 2017 (*Figure 1A* and *B*), with a long segment of thrombus, treated by thrombus aspiration and angioplasty with a stent. Clopidogrel was initiated in addition to enoxaparin. His other regular medications were ramipril, bisoprolol, atorvastatin, and metformin. He was referred to the haematology team for suspected thrombophilia, which was ruled out after a thorough assessment (negative screen).

The transthoracic echocardiography at that point revealed a well-seated metallic aortic valve replacement (AVR) with no AR and no evidence of thrombus. After 1 month, he developed a small trochanteric haematoma after a fall, and clopidogrel was discontinued whereas enoxaparin monotherapy continued. In April 2019, he presented to the clinic with exertional breathlessness, orthopnoea, and paroxysmal nocturnal dyspnoea. He was compliant with medication. Clinical investigation revealed signs of heart failure and a harsh ejection systolic murmur with an audible second mechanical click. He was stable haemodynamically (blood pressure: 121/78 mmHg; heart rate: 53 b.p.m.; respiratory rate: 20 cycles/minute; oxygen saturation in the room air: 98%).

**Figure 2:**
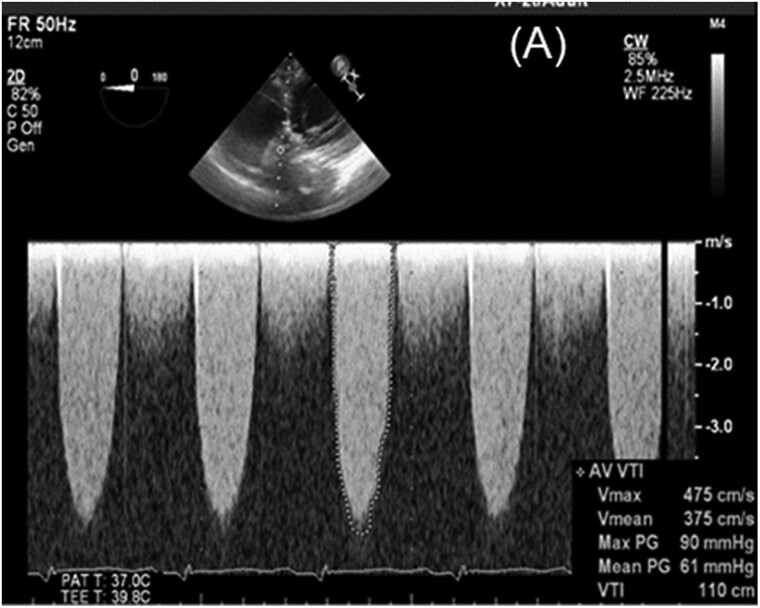
Trans-oesophageal echocardiogram demonstrating severe aortic obstruction.

**Figure 3:**
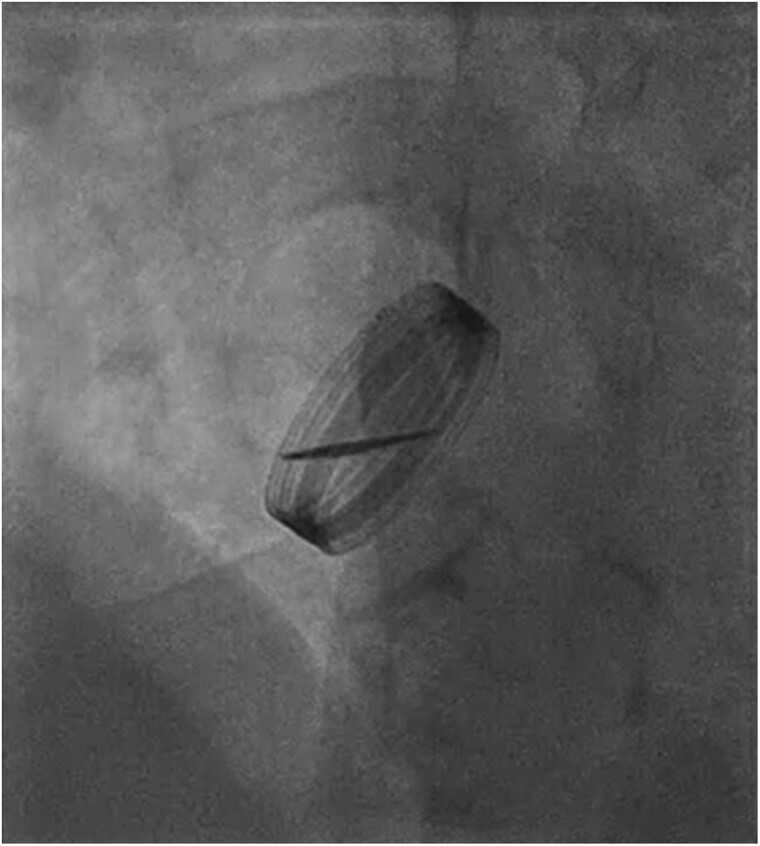
Prosthetic valve fluoroscopy indicating a fixed immobile disc that obstructs blood flow.

**Figure 4:**
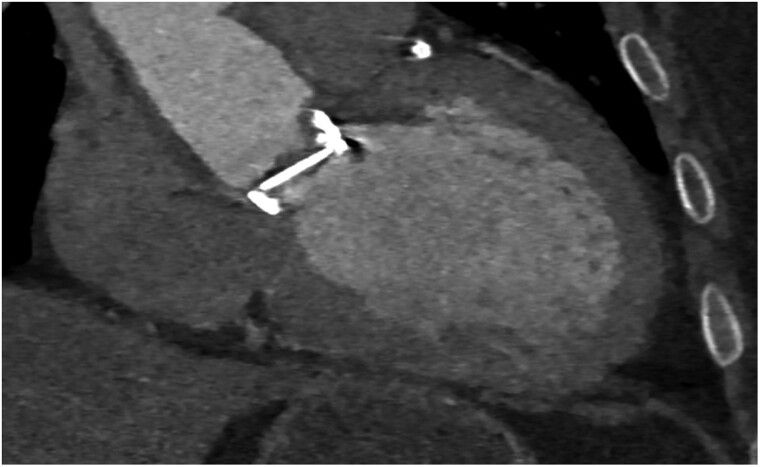
Computed tomography aortogram showing pannus and thrombus in the aortic surface of the metallic aortic prosthesis.

An electrocardiogram revealed a first-degree heart block with no new ST or T-wave changes. A chest X-ray detected bilaterally increased pulmonary vascular markings with septal lines and features, which would be most consistent with cardiac failure. A transthoracic echocardiogram demonstrated moderate to severe left ventricular function impairment [visually estimated ejection fraction (EF): 35–40%]. Evidence indicates severe aortic stenosis with a peak velocity of 3.9 m/s. Aortic valve peak gradient was 59 mmHg, and AV mean gradient was 41 mmHg, with no evidence of pulmonary hypertension. We ruled out a thrombus on mechanical valve leaflets. He was admitted for inpatient investigations. An urgent trans-oesophageal echocardiogram revealed a mean gradient of 61 mmHg and a peak velocity of 4.75 m/s (*Figure 2*). Valve fluoroscopy revealed a fixed immobile leaflet that obstructs blood flow (*Figure 3*) (see Supplementary material online, *Videos S1* and *S2*). No laboratory evidence indicated haemolysis, haemoglobin was 139 g/L (normal range: 115–160 g/L), bilirubin was 7 µmol/L (normal range: 0–20 µmol/L), troponin was negative, C-reactive protein was <5 mg/L (normal range: 0–105 mg/L), and total white cell count was 6.0 × 10^9^/L (normal range: 4.00–11.00 × 10^9^/L). Clotting profiles were within the normal limit, including PT of 11.2 s (normal range: 10–13 s), activated partial thromboplastin time of 27.3 s (normal range: 30–40 s), thrombin time of 15.3 s (normal range: 12–19 s), fibrinogen of 2 g/L (normal range: 2–4.5 g/L), B-type natriuretic peptide of 469.30 ng/L (normal range: 0.00–100 ng/L), creatinine of 142 µmol/L (normal range: 59–104 µmol/L), and estimated glomerular filtration rate of 48 mL/min/1.73 m (normal range: 60–200 mL/min/1.73 m). A computed tomography aortogram revealed the pannus around the metallic aortic valve (*Figure 4*). The decision after discussion in the multidisciplinary team was against thrombolytic agents, including low-dose tissue plasminogen activator infusion or thrombolysis, considering a history of three previous stroke episodes.

Intravenous heparin infusion and diuretics were initiated, and serial echocardiography detected reductions in gradient. The repeated transthoracic echocardiogram revealed one fixed disc, with a mean gradient of 61 mmHg, peak velocity of 3.63 m/s, annulus of 2.1 cm, AVA of 0.3 cm^2^, and severely impaired left ventricular function (visually estimated EF of <35%). However, leaflet function exhibited no clinical improvement or change over a 3- to 4-week period with medical therapy. The multidisciplinary team recommended a redo AVR, which the patient accepted.

**Figure 5:**
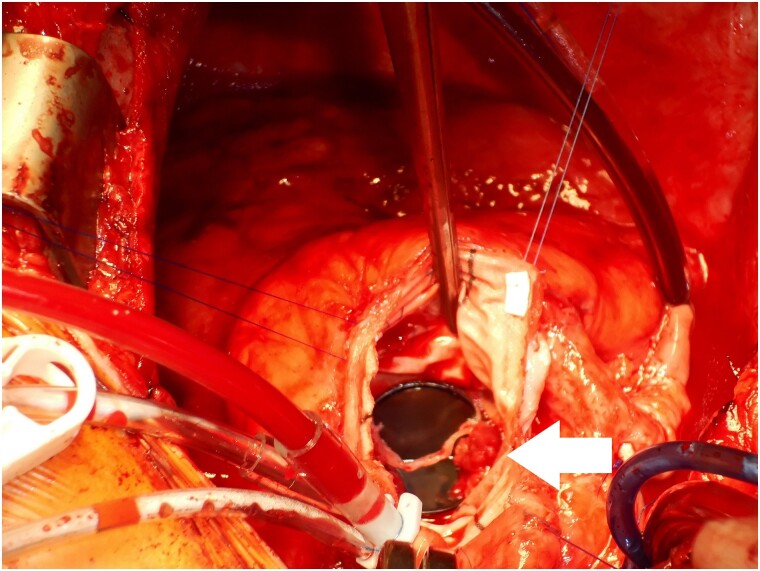
Intra-operative surgeon’s view of the mechanical leaflet with pannus (white arrow).

Intra-operative results indicated an immobile mechanical leaflet due to pannus formation both above and below the left coronary cusp leaflet, as well as a fixed, organized thrombus that obstructs the flow and valve tilting (*Figure 5*). The patient received a biological valve (25 mm of INSPIRIS RESILIA bioprosthetic valve), with excellent post-operative recovery and free from further embolic events and symptoms.

## Discussion

The patient in this case report was given subcutaneous enoxaparin (LMWH) as an anticoagulant a year after his metallic valve replacement. Warfarin therapy had failed due to erratic INR and minor bleeding episodes. Regular monitoring of factor Xa level was not performed in our patient despite multiple episodes of thromboembolic events. Eventually, he had symptoms of mechanical valve failure 12 years after mechanical AVR, which required redo surgery.

Bleeding episodes and thromboembolic events are the most prevalent complications encountered in patients with mechanical heart valves. Thromboembolic events depend on many factors, including age, atrial fibrillation, left ventricular function, kidney function, body weight, and compliance with anticoagulants. Self-INR monitoring has revealed consistent achievement of target levels and decreased major bleeding events.^7^ However, challenges include the monitoring of INR, labile INR, and VKA intolerance. Subcutaneous LMWH is an alternative to UFH for bridging to a therapeutic INR, which is administered twice daily with a therapeutic dose adjusted by body weight, considering alternatives to VKAs in these patients. The administration should be guided by anti-Xa activity monitoring with a target of 0.5–1.0 U/mL.^8^ Strict anti-Xa monitoring and therapeutic dose compliance with enoxaparin during pregnancy reduced the risk of valve thrombosis and good foetal outcomes.^9^ However, the outcomes are variable. The role of long-term LMWH is associated with the risk of decreased bone density and fracture as demonstrated in our case.^10^ Our patient received LMWH of enoxaparin at 1 mg/kg administered twice daily, a year after his mechanical AVR operation, following erratic INR fluctuation 1–6 with minor bleeding episodes (epistasis).

Anti-factor Xa levels are not regularly monitored in our institution unless patients are underweight, obese, or pregnant or with renal impairment (creatinine clearance < 30 mL/min). Introducing direct oral anticoagulant—dabigatran—should be considered, but it was not started due to uncertain benefits and because more bleeding and thromboembolic complications were encountered (RE-ALIGN trial 2014—Randomized, Phase II Study to Evaluate the Safety and Pharmacokinetics of Oral Dabigatran Etexilate in Patients after Heart Valve Replacement). Therefore, the patient was continued on LMWH for his mechanical heart valve. He experienced multiple thromboembolic episodes, both stroke and STEMI, while on enoxaparin, and ultimately developed symptoms of mechanical valve failure 12 years after metallic AVR, which required redo surgery (replacement with tissue AVR).

Cases of varying outcomes associated with long-term prescriptions of anticoagulants for patients with mechanical aortic valves have been reported. A 31-year-old male patient with metallic AVR survived for 31 years without an antithrombotic treatment before failing valve function.^11^

A 43-year-old male patient with 6 months post-metallic AVR without anticoagulation, who had presented with cardiogenic shock, required emergency surgical thrombectomy.^12^ Long-term anticoagulation with LMWH in a 72-year-old male patient who had a mechanical aortic valve was successful without major bleeding risks and thromboembolic episodes for 13 years.^13^ A patient with atrial fibrillation and double mechanical valves who was prescribed twice a day of apixaban 5 mg demonstrated no thromboembolic events after 2 years of observation.^14^ The introduction of long-term LMWH for mechanical aortic valves was unsuccessful in our case. Regular monitoring of anti-factor Xa levels would be useful, but more research is required concerning standardized guidance.

## Conclusions

Vitamin K antagonists are the only proven lifelong anticoagulants for all patients with mechanical heart valves. Management of patients with VKA intolerance is challenging. Long-term anticoagulation with LMWH may be an alternative to warfarin for those who are intolerant to VKAs. However, anti-factor Xa level monitoring may be useful. Further randomized controlled studies are required to establish the efficient and safe use of LMWH in patients with mechanical heart valves. This reinforces the importance of counselling the patient regarding anticoagulation when selecting between mechanical and biological prostheses.

## Lead author biography


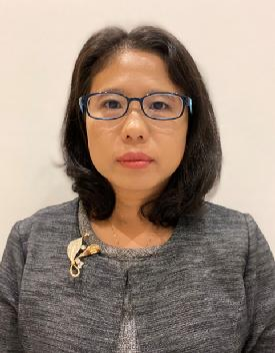


Dr Aye Aye Mon is a cardiology trainee under Health Education England.

## Supplementary Material

ytae361_Supplementary_Data

## Supplementary material

**Consent:** Written informed consent was obtained from the patient to publish this report and accompanying images in line with the COPE guidelines.

**Funding:** None declared.

### Data availability

The data underlying this article are available in the article and in its online Supplementary material.

## References

[1] Roudaut R , Serri K, Lafitte S. Thrombosis of prosthetic heart valves: diagnosis and therapeutic considerations. Heart 2007;93:137–142. 17170355 10.1136/hrt.2005.071183 PMC1861363

[2] Cannegieter SC , Rosendaal FR, Briët E. Thromboembolic and bleeding complications in patients with mechanical heart valve prostheses. Circulation 1994;89:635–641. 8313552 10.1161/01.cir.89.2.635

[3] Caldeira D , David C, Santos AT, Costa J, Pinto FJ, Ferreira JJ. Efficacy and safety of low molecular weight heparin in patients with mechanical heart valves: systematic review and meta-analysis. J Thromb Haemost 2014;12:650–659. 24593838 10.1111/jth.12544

[4] Greiten LE , Mckellar SH, Rysavy J, Schaff HV. Effectiveness of rivaroxaban for thromboprophylaxis of prosthetic heart valves in a porcine heterotopic valve model. Eur J Cardiothorac Surg 2014;45:914–919. 24306948 10.1093/ejcts/ezt545

[5] Ribeiro PA , Al Zaibag M, Idris M, Al Kasab S, Davies G, Mashat E, et al Antiplatelet drugs and the incidence of thromboembolic complications of the St. Jude Medical aortic prosthesis in patients with rheumatic heart disease. J Thorac Cardiovasc Surg 1986;91:92–98. 3941564

[6] Wenos CD , Herrmann JL, Timsina LR, Patel PM, Fehrenbacher JW, Brown JW. Perioperative and long-term outcomes of Ross versus mechanical aortic valve replacement. J Card Surg 2022;37:2963–2971. 35989510 10.1111/jocs.16831 PMC9542516

[7] Chen QL , Dong L, Dong YJ, Zhao SL, Fu B, Wang YQ, et al Security and cost comparison of INR self-testing and conventional hospital INR testing in patients with mechanical heart valve replacement. J Cardiothorac Surg 2015;10:4. 25592732 10.1186/s13019-015-0205-1 PMC4308889

[8] Vahanian A , Alfieri O, Andreotti F, Antunes MJ, Barón-Esquivias G, Baumgartner H, et al Guidelines on the management of valvular heart disease (version 2012). Eur Heart J 2012;33:2451–2496. 22922415 10.1093/eurheartj/ehs109

[9] McLintock C , McCowan LME, North RA. Maternal complications and pregnancy outcome in women with mechanical prosthetic heart valves treated with enoxaparin. BJOG 2009;116:1585–1592. 19681850 10.1111/j.1471-0528.2009.02299.x

[10] Wawrzyńska L , Tomkowski WZ, Przedlacki J, Hajduk B, Torbicki A. Changes in bone density during long-term administration of low-molecular-weight heparins or acenocoumarol for secondary prophylaxis of venous thromboembolism. Pathophysiol Haemost Thromb 2003;33:64–67. 10.1159/000073848 14624046

[11] Yalçın M , Özkan H, Tiryakioğlu O. A 31-year-old patient without the use of warfarin and with an aortic mechanical valve. Anatol J Cardiol 2017;17:494–495. 28617299 10.14744/AnatolJCardiol.2017.7853 PMC5477086

[12] De la Rocha AG , Plume SK, Baird RJ. Thrombosis of Bjork–Shiley aortic valve prosthesis: report of three cases. Can Med Assoc J 1977;116:137–142. PMC1879496 861869

[13] Wang X , Mathew C, Korapati S, Bathini VG. Successful long-term anticoagulation with enoxaparin in a patient with a mechanical heart valve. Pharmacotherapy 2020;40:174–177. 31885093 10.1002/phar.2361

[14] Eom JY , Shin JK, Kwon CH. Apixaban use in an atrial fibrillation patient with double mechanical heart valves: a case report. Eur Heart J Case Rep 2021;5:ytab285. 34377918 10.1093/ehjcr/ytab285 PMC8343445

